# Effects of curcumin on ion channels and transporters

**DOI:** 10.3389/fphys.2014.00094

**Published:** 2014-03-11

**Authors:** Xuemei Zhang, Qijing Chen, Yunman Wang, Wen Peng, Hui Cai

**Affiliations:** ^1^Department of Pharmacology, School of Pharmacy, Fudan UniversityShanghai, China; ^2^Department of Nephrology, Putuo Hospital, Shanghai University of Traditional Chinese MedicineShanghai, China; ^3^Renal Division, Department of Medicine, Department of Physiology, Emory University School of MedicineAtlanta, GA, USA; ^4^Section of Nephrology, Atlanta Veterans Administration Medical CenterDecatur, GA, USA

**Keywords:** curcumin, ion channels, drug targets, transporters, membrane

## Abstract

Curcumin [1,7-bis(4-hydroxy-3-methoxyphenyl)-1,6-heptadiene-3,5-dione], a polyphenolic compound isolated from the rhizomes of Curcuma longa (turmeric), has been shown to exhibit a wide range of pharmacological activities including anti-inflammatory, anti-cancer, anti-oxidant, anti-atherosclerotic, anti-microbial, and wound healing effects. These activities of curcumin are based on its complex molecular structure and chemical features, as well as its ability to interact with multiple signaling molecules. The ability of curcumin to regulate ion channels and transporters was recognized a decade ago. The cystic fibrosis transmembrane conductance regulator (CFTR) is a well-studied ion channel target of curcumin. During the process of studying its anti-cancer properties, curcumin was found to inhibit ATP-binding cassette (ABC) family members including ABCA1, ABCB1, ABCC1, and ABCG2. Recent studies have revealed that many channels and transporters are modulated by curcumin, such as voltage-gated potassium (Kv) channels, high-voltage-gated Ca^2+^ channels (HVGCC), volume-regulated anion channel (VRAC), Ca^2+^ release-activated Ca^2+^ channel (CRAC), aquaporin-4 (AQP-4), glucose transporters, etc., In this review, we aim to provide an overview of the interactions of curcumin with different types of ion channels and transporters and to help better understand and integrate the underlying molecular mechanisms of the multiple pharmacological activities of curcumin.

## Introduction

Turmeric, the rhizome of *Curcuma longa L*. has been used since ancient times as a spice, coloring, flavoring, and traditional medicine. Curcumin [1,7-bis(4-hydroxy-3-methoxyphenyl)-1,6-heptadiene-3,5-dione], an important active agent of turmeric, has been shown to exhibit a wide range of pharmacological activities including anti-inflammatory, anti-cancer, anti-oxidant, anti-atherosclerotic, anti-microbial, and wound healing effects (Maheshwari et al., 2006). These activities of curcumin are based on its chemical features, as well as its ability to interact with multiple signaling molecules. Many biological molecules have been identified as targets of curcumin, including transcription factors, growth factors, inflammatory cytokines, protein kinases and other enzymes (Zhou et al., 2011). The safety, tolerability, and non-toxicity of curcumin at high doses have been well established in many clinical trials. Different phase I clinical trials indicated that curcumin is well tolerated when taken in dose as high as 12 g per day. However, curcumin exhibits poor bioavailability at the same time (Anand et al., 2007). Thus, numerous approaches have been undertaken to improve its bioavailability, including the use of piperine, nanoparticles, liposomes, phospholipid complexes, and structural analogues (Gupta et al., 2013).

Studies have shown that curcumin influences a variety of ion channels and transporters across several signaling pathways, ranging from the well-studied CFTR to recently identified hERG potassium channel. The list is continuously expanding and it would not be surprising to find novel molecules in the future. Here, we summarized the known effects of curcumin on ion channels and transporters to help better understanding the underlying molecular mechanisms of multiple pharmacological activities of curcumin.

## Effects of curcumin on ion channels

### Potassium channels

Kv channels play important roles in regulating resting membrane potential. It has long been observed that cells possessing a high degree of polarization tend to be quiescent (Wonderlin and Strobl, 1996; Sundelacruz et al., 2009). As a potent blockage of Kv channels, curcumin reduces the Kv current in rabbit coronary arterial smooth muscle cells (Hong Da et al., 2013) and Jurkat T cells (Shin et al., 2011), inhibits Kv1.3 in effector memory T cells (TEM) (Lian et al., 2013), and blocks Kv11.1 potassium current in human monocytic leukemia (AML) cell lines THP-1 (Banderali et al., 2011) and HEK293 (Choi et al., 2013). Curcumin prevents Kv channel from activating, which consequently suppresses proliferation of AML cells and TEM cells, whereby exerts its anti-cancer or anti-inflammatory effects (Banderali et al., 2011; Lian et al., 2013). However, the mechanisms underlying the inhibitory effects of curcumin are not fully understood. Several studies suggest that curcumin inhibits Kv current via direct action on the Kv channels (Choi et al., 2013; Hong Da et al., 2013; Lian et al., 2013), possibly through its interaction with the pore blocker binding site (Choi et al., 2013).

Curcumin is also an effective antinociceptive agent. Studies suggest that activation of K_ATP_ channels, possibly by direct stimulation, contributes to the antinociceptive effect of curcumin (De Paz-Campos et al., 2012). Additional potential mechanisms of the antinociceptive effect of curcumin include activating G_*i/o*_ proteins, stimulating the particular form of guanylyl cyclase or acting through the hydrogen sulfide-KATP channel pathway. Since the direct evidence is still lacking, further study is needed to fully reveal this mechanism. Curcumin has also been shown to inhibit intermediate-conductance Ca^2+^-activated K^+^ channel (SK4) (Shin et al., 2012), but relevant physiological study is still elusive.

On the other hand, curcumin has been shown to open K^+^ channel. In goat ruminal artery, curcumin induces vasorelaxation by, at least in part, directly activating soluble guanylate cyclase (sGC) mediated cGMP pathway followed by the opening of K^+^ ion channel (Dash and Parija, 2013). Given the diverse regulatory effects of curcumin on multiple targets, it is not surprising that curcumin may participate in different pathways and lead to different physiological actions of K^+^ channels.

### Calcium channels

The increase of the intracellular Ca^2+^ concentration is widely viewed as the most important contributor to neurodegeneration and neuronal cell death (Duncan et al., 2010). Calcium channel blockade is one approach among the neuroprotective strategies (Singer, 2012). Curcumin has been demonstrated to reversibly inhibit HVGCC currents via a novel protein kinase C-θ -dependent pathway, which could contribute to its neuroprotective effects in rat hippocampal neurons (Liu et al., 2013). In another study, it has been shown that curcumin inhibits glutamate release from rat prefrontocortical synaptosomes by suppressing presynaptic voltage-gated calcium channels Cav2.2 and Cav2.1 (Lin et al., 2011). This effect of curcumin might relate to the mechanisms underlying the antidepressant effect of curcumin. Curcumin has also been observed to inhibit Ca^2+^ release-activated Ca^2+^ (CRAC) channels (Shin et al., 2011, 2012).

Calcium influx is mainly mediated by store-operated Ca^2+^ entry (SOCE) through CRAC channels located in the plasma membrane, which is important for the activation and function of all cells in the immune system (Shaw et al., 2012). The inhibition of CRAC and another major ion channel Kv1.3 in lymphocytes might contribute to the anti-inflammatory effect of curcumin. The function of CRAC channels is primarily mediated by Orai proteins, which are located in the plasma membrane as the Ca^2+^ conducting pore unit (Shaw et al., 2012). Curcumin contains electrophilic α,β -unsaturated carbonyl groups that potentially form Michael addition with cysteine residues. The electrophilic addition to the Orai1 195Cys is responsible for the inhibitory effect of CRAC by curcumin (Choi et al., 2013).

### Chloride channels

CFTR acts as a Cl^−^ channel on the apical membrane of epithelia. Mutations in the CFTR gene cause the reduction of CFTR expression or abnormalities in its function, thereby resulting in cystic fibrosis (CF), a genetic disease. The most common CF-causing mutation is ΔF508-CFTR, which leads to CFTR protein misfolding and retention in the endoplasmic reticulum (ER). Wild type and mutant CFTR channels can be activated by curcumin. Curcumin not only rescues ΔF508-CFTR localization by allowing ΔF508-CFTR to escape from ER and to anchor in the plasma membrane (Egan et al., 2004; Cartiera et al., 2010), but also stimulates its channel activity once it reaches the plasma membrane (Berger et al., 2005; Lipecka et al., 2006). This capacity of curcumin to release ΔF508-CFTR from the ER is likely due to the dissolution of the calnexin-ΔF508-CFTR complex and the stabilization of the tertiary structure ofΔF508-CFTR (Egan et al., 2004). Curcumin also potentiates another CF mutant channel, the glycine-to-aspartate missense mutation at position 551 (G551D). G551D-CFTR is the third most common CF-associated mutation, which is characterized by an extremely low open probability despite its normal trafficking to the plasma membrane (Miki et al., 2010). Curcumin increases the activity of the G551D-CFTR mutant channel (Yu et al., 2011). The cross-link of curcumin and these two CF mutant channels could be prevented by high concentrations of oxidant scavengers *In Vitro*, indicating a possible oxidation reaction of curcumin with the CFTR polypeptide (Bernard et al., 2009).

However, some studies fail to reproduce these results. Song et al. are unable to demonstrate the effectiveness of curcumin on the functional correction of the defective ΔF508-CFTR processing in transfected cells, native airway cells, and mutant mice (Song et al., 2004). Dragomir et al. have observed that curcumin causes a small increase in net cAMP-activated chloride efflux from ΔF508-CFTR expressing baby hamster kidney (BHK) cells, but fails to show the significant movements of ΔF508-CFTR to the plasma membrane in ΔF508-CFTR BHK cells (Dragomir et al., 2004). Gao et al. have shown that curcumin does not change CFTR expression level and forskolin-induced CFTR gating in Madin–Darby canine kidney (MDCK) cell monolayer (Gao et al., 2011). These contradictions may be due to different genetic backgrounds of the animals studied, or different preparation of curcumin extract, etc. (Mall and Kunzelmann, 2005). Thus, although curcumin may be a desirable alternative for treating CF, its efficacy needs to be carefully evaluated (Mall and Kunzelmann, 2005).

VRAC plays an important role in regulating electrical and secretory activity in the β -cell. Curcumin activates VRAC, leading to Cl^−^ efflux in β -cells. A study using single channel recording has indicated that this activation is the result of increased channel open probability (Gao et al., 2011). This study could partly explain the hypoglycemic action of curcumin.

### Other channels

AQP-4, the predominant isoform of water channels in the brain, plays an important role in fluid generation, transfer, and absorption in brain. Curcumin attenuates the increase of AQP-4 expression induced by hypoxic–ischemic brain damage (HIBD) in the hippocampus (Yu et al., 2012). Since blood-brain barrier opening depends on the upregulation of AQP-4 function (Papadopoulos and Verkman, 2007), this study suggests that by down-regulating AQP-4, curcumin may protect astrocytic foot processes surrounding brain capillaries from damaging from HIBD edema. Curcumin could also block IL-1β -induced AQP-4 expression in the cultured astrocytes and further reduce glial activation and cerebral edema following neurotrauma (Laird et al., 2010). However, in lupus erythematosus, curcumin increases brain AQP4 expression and water content (Foxley et al., 2013). Along with other evidence, it seems that curcumin aggravates some CNS disease manifestations in experimental lupus erythematosus.

Transient receptor potential (TRP) cation channel subfamily A member 1 (TRPA1) is a nociceptor specific ion channel expressed in a subset of TRPV1-expressing neurons. It has been an attractive target for various therapeutical interventions in the disease conditions, such as pain, asthma, and cough (Bandell et al., 2004). Curcumin causes activation and subsequent desensitization of native and recombinant TRPA1 ion channels of multiple mammalian species (Leamy et al., 2011). This effect may contribute to the analgesic effect of curcumin in patients with various chronic diseases (Di Pierro et al., 2013).

Curcumin is also a potent inhibitor of the inositol 1,4,5-trisphosphate-sensitive Ca^2+^ channel (IP3 receptor). By inhibiting IP3 receptor, curcumin stimulates Ca^2+^ uptake, reduces Ca^2+^ leakage and inhibits IP3-induced Ca^2+^ release (IICR) from ER Ca^2+^ storage, blocks a multitude of subsequent Ca^2+^-dependent cellular events. This inhibition is likely non-competitive in nature (Dyer et al., 2002).

## Effects of curcumin on transporters

### ATP-binding cassette (ABC) drug transporter

ABC proteins are a large family of integral membrane proteins. They, collectively, serve a wide variety of cellular functions. ABC drug transporters actively transport a variety of amphipathic compounds. The overexpression of ABC drug transporters causes multidrug resistance (MDR) in cancer cells. Among ABC transporter family, three of them, ABCB1 (P-glycoprotein/Pgp), ABCC1 (multidrug resistance protein 1/MRP1) and ABCG2 (breast cancer resistance protein /MXR/BCRP) appear to play an important role in the development of MDR in cancer cells (Wu et al., 2008; Orina et al., 2009). Curcumin has been studied as a potential anticancer drug for many years, not only because of its antiproliferative or apoptosis-inducing effect on several cancer cells, but also due to its ability to reverse MDR phenotypes in several cancer cells overexpressing ABC transporters, such as, ABCB1, ABCC1, and ABCG2 (Sharma et al., 2009). Curcuminoids (including curcumin, demethoxycurcumin and bisdemethoxycurcumin) sensitizes the ABCG2-expressing cells to conventional chemotherapeutic agent mitoxantrone, topotecan, SN-38, and doxorubicin. The reversal of resistance is due to their influence on the function rather than the protein levels of ABCG2 (Chearwae et al., 2006). Curcumin also inhibits ABCG2-mediated efflux of sulfasalazine and increases the area under the curve (AUC) of plasma sulphasalazine both *in vivo* (Shukla et al., 2009) and *in vitro* (Kusuhara et al., 2012). In the case of ABCB1 (Pgp), its overexpression on the surface of tumor cells is often linked to MDR. In the multidrug-resistant human cervical carcinoma cell line KB-V1, curcumin is able to significantly lower the Pgp expression and reduce the function of Pgp. Curcumin is not a substrate for Pgp, but it interacts directly with drug binding site of the transporter (Anuchapreeda et al., 2002; Limtrakul et al., 2007). Treatment of KB-V1 cells with curcumin increases their sensitivity to vinblastine (Anuchapreeda et al., 2002; Limtrakul et al., 2007). In MCF-7/ADR cells overexpressing Pgp, curcumin significantly enhances the cellular accumulation of rhodamine-123 in a concentration-dependent manner, indicating that curcumin significantly inhibits Pgp activity (Cho et al., 2012). This evidence suggests that curcumin could be an effective MDR modulator, and may be used in combination with conventional chemotherapeutic drugs to reverse MDR in cancer cells.

Needless to say, the trafficking of curcumin depends on transporters as well. Curcumin-resistance has also been observed in some cancer cells, and efflux of curcumin by ABC transporters is considered as one of the factors causing resistance. In hypoxia-induced curcumin-resistance in HepG2 cells, the expression of ABC drug transporter genes, including ABCC1, ABCC2, and ABCC3, are increased by more than two-fold. Both inhibitors of ABCC1/ABCC2 are able to reverse this curcumin resistance (Sakulterdkiat et al., 2012). In resistant M14 melanoma cells, the ABCA1 is over-expressed as compared to that in the curcumin-sensitive MDA-MB-231 breast cancer cells. Gene silencing of ABCA1 sensitizes M14 cells to the apoptotic effect of curcumin (Bachmeier et al., 2009). Therefore, the expression level of the involved ABC drug transporters should be monitored as potential response predictors in curcumin treatment for certain types of cancer.

ABC molecules, especially ABCA and ABCG subfamily members, are critical in the regulation of lipid-trafficking as well (Schmitz et al., 2001), through which curcumin affects lipid metabolism, contributing its beneficial effects on inflammation, cardiovascular diseases and obesity. In macrophages, treatment with curcumin markedly ameliorates cholesterol accumulation by increasing cholesterol efflux, due to up-regulation of ABCA1. Curcumin administration modulates the expression of ABCA1 and ABCG1 in apoE^−/−^ mice (Zhao et al., 2012). Curcumin also increases the cholesterol efflux from adipocytes. The increased expressions of PPARγ, LXRα and ABCA1 induced by curcumin are parallel and correlated, suggesting that curcumin can affect the cholesterol efflux from adipocytes by regulating the PPARγ-LXR-ABCA1 pathway (Dong et al., 2011).

### Glucose transporter (GLUT)

One of the merits for curcumin used as an anti-diabetic medication is its ability to lower blood glucose likely by interacting with GLUT. Evidence suggests curcumin influences subtypes of GLUT. Hyperglycemia stimulates hepatic stellate cell (HSC) activation *in vitro* by increasing intracellular glucose. Curcumin eliminates this stimulatory effect via blocking the membrane translocation of GLUT2 and suppressing GLUT2 expression. The former effect is mediated by interrupting the p38 MAPK signaling pathway and the latter by activating PPARγ and attenuating oxidative stress (Lin and Chen, 2011). In the leptin-induced HSC activation, curcumin suppresses the membrane translocation of GLUT4 by interrupting the insulin receptor substrates (IRS)/PI3K/AKT signaling pathway (Tang and Chen, 2010). On the other hand, Cheng et al. have shown that curcumin causes a concentration-dependent increase of glucose uptake by skeletal muscle cells isolated from Wistar rats, and this action is mediated by increasing membrane protein level of GLUT4 (Cheng et al., 2009). The increased GLUT4 is reversed by blocking muscarinic M-1 cholinoceptor (M_1_-mAChR) or PLC/PI3K pathway. These phenomena suggest that the effects of curcumin on GLUT are complicated and may be tissue specific and/or signaling pathway-dependent.

### Other transporters

Glutamate facilitates the pathogenesis of post-ischemic neuronal injury. Glutamate transporter-1 (GLT-1) is essential for maintaining a low extracellular glutamate concentration and for preventing glutamate neurotoxcity. Curcumin may reduce cerebral vasospasm (CVS) and neurologic injury via an antioxidant effect and attenuate glutamate-induced neurotoxicity. In a subarachnoid hemorrhage (SAH)-induced rat CVS model, glutamate levels are lower in the curcumin treated group vs. the saline and vehicle treated groups. Correspondingly, GLT-1 is preserved after SAH in curcumin-treated rats (Kuo et al., 2011). These results suggest that curcumin may modulate GLT-1.

Curcumin increases the expression of two lipid transport genes, the fatty acids transporter CD36/FAT and the fatty acids binding protein 4 (FABP4/aP2) in THP-1 and RAW264.7 monocytes and macrophages, leading to increased lipid levels in THP-1 and RAW264.7 (Zingg et al., 2012), though the significance of the findings on the effect of curcumin against oxidant and lipid-induced damage needs to be further studied.

Intestinal Niemann-Pick C1-like 1 (NPC1L1) cholesterol transporter plays an essential role in the maintenance of cholesterol homeostasis. Curcumin exhibits effects of lowering plasma cholesterol and preventing diet-induced hypercholesterolemia. Treating Caco-2 cell monolayers with curcumin significantly inhibits cholesterol esterification and uptake. Coincidentally, the NPC1L1 mRNA level and protein expression are significantly decreased (Feng et al., 2010; Kumar et al., 2011). This evidence suggests that hypocholesterolemic effect of curcumin may be linked to the suppression of NPC1L1 expression in the intestinal cells.

### Mechanisms underlying regulatory effects of curcumin

Although it has been under intensive investigations and scientific debates, the detailed mechanisms underlying the diverse effects of curcumin remain elusive. One argument is that curcumin is a modulator of membrane structure; it may affect the membrane proteins in a non-specific way. Ingolfsson et al. explored whether curcumin modifies general lipid bilayer properties using channels formed by gramicidin A (gA). They found that curcumin decreases the stiffness of the lipid-bilayer and increases the lifetimes and appearance rates of gA channel, suggesting that the energetic cost of the gA-induced bilayer deformation is reduced (Ingolfsson et al., 2007). Another study showed that curcumin affects membrane structure in a manner analogous to lipophillic drugs, which are inserted deeply into the membrane in a transbilayer orientation and anchored by hydrogen bonding to the phosphate group of lipids (Barry et al., 2009). Other studies, however, demonstrated that curcumin possesses a specific property or mechanism underlying its modulation of the transporters or channels. In a case of CFTR regulation, curcumin is able to cross-link CFTR polypeptides and phosphorylate the R domain of CFTR channel (Bernard et al., 2009). For the hERG potassium channel, curcumin exerts the pore-blocking effect (Choi et al., 2013). The effect of curcumin on TRPV1 channel is dependent on disease conditions. Curcumin exerts no effects on the TRPV1 channel in normal tissue, whereas activates TRPV1 channel in colitis (Martelli et al., 2007). These studies suggest that curcumin exhibits its unique effects on ion channels and transporters through different mechanisms far beyond a non-specific effect although some of curcumin's effectiveness may be membrane-mediated. The exact mechanisms on each particular channel and transporter need to be further elucidated.

## Conclusion

These studies have provided ample evidence that curcumin affects a variety of ion channels and transporters, and hence modulates various critical physiological functions. In many circumstances, regulatory effects of curcumin may be presented with a multi-faceted nature given the multiple targets and different pathways in which curcumin is involved. Despite there being several clinical trials involving curcumin, no clear clinical indication has been yet defined. More studies are needed to fully evaluate the efficacy of curcumin in its utility as a therapeutic agent for the different diseases. Nevertheless, given its potential multiple targets, pharmacological safety and enhanced bioavailability, curcumin is a promising natural product that is likely to be brought to the forefront of therapeutic agents for treatment of human diseases in the future.

### Conflict of interest statement

The authors declare that the research was conducted in the absence of any commercial or financial relationships that could be construed as a potential conflict of interest. The Associate Editor He-Ping Ma declares that, despite being affiliated to the same institution as the author Hui Cai, the review process was handled objectively and no conflict of interest exists.
